# Development of an Enzyme Linked Immunosorbent Assay and an Immunochromatographic Assay for Detection of Organophosphorus Pesticides in Different Agricultural Products

**DOI:** 10.1371/journal.pone.0053099

**Published:** 2012-12-31

**Authors:** Xiude Hua, Jifei Yang, Limin Wang, Qingkui Fang, Gaiping Zhang, Fengquan Liu

**Affiliations:** 1 College of Plant Protection, Nanjing Agricultural University, Nanjing, China; 2 Key Laboratory of Integrated Management of Crop Diseases and Pests, Ministry of Education, Nanjing, China; 3 Henan Provincial Key Laboratory for Animal Immunology, Henan Academy of Agriculture Sciences, Zhengzhou, China; Cordelier Research Center, INSERMU872-Team16, France

## Abstract

**Objective:**

Organophosphorus (OP) pesticides are considered hazardous substances because of their high toxicity to nontarget species and their persistence in the environment and agricultural products. Therefore, it is important to develop a rapid, sensitive, and economical method for detecting OP pesticides and their residues in food and the environment.

**Methods:**

A broad, selective monoclonal antibody (MAb) for organophosphorus pesticides was produced. Based on the MAb, an enzyme linked immunosorbent assay (ELISA) and an immunochromatography assay (ICA) for detecting OP pesticides in different agricultural products were developed using a binding inhibition format on microtiter plates and a membrane strip, respectively.

**Results:**

Under the optimized conditions, the IC_50_ values of the ELISA ranged from 3.7 to 162.2 ng mL^–1^ for the 8 OP pesticides. The matrix interferences of Apple, Chinese cabbage, and greengrocery were removed by 40-fold dilution, the recoveries from spiked samples ranged from 79.1% to 118.1%. The IC_50_ values of ICA for the 8 OP pesticides ranged from 11.8 to 470.4 ng mL^−1^. The matrix interference was removed from the Chinese cabbage and Apple samples with 5-fold dilution, and the interference was removed from the greengrocery samples with 20-fold dilution. The recoveries from the spiked samples ranged between 70.6 and 131.9%. The established ELISA and ICA were specific selectivity for the 8 OP pesticides.

**Conclusions:**

The established ELISA is a sensitive screening method for the detection of OP pesticides, but the ELISA detection method depends on a laboratory platform and requires a relative long assay time and several steps operation. The established ICA is very useful as a screening method for the quantitative, semi-quantitative or qualitative detection of OP pesticides in agricultural products, and it has advantages over ELISA methods with regard to factors such as the testing procedure, testing time, and matrix interferences, among others.

## Introduction

Organophosphorus (OP) pesticides are widely used in agriculture for sucking and biting insect pest control, including fruit flies, stem borers, mosquitoes, and *Eurygaster* cereal bugs. However, OP pesticides are considered hazardous substances because of their high toxicity to nontarget species and their persistence in the environment [1]. Additionally, there is increasing concern over food and environmental contamination resulting from the overuse of pesticides [2]. Therefore, it is important to develop a rapid, sensitive, and economical method for detecting OP pesticides and their residues in food and the environment.

Currently, instrument-based methods, such as gas chromatography and high-performance liquid chromatography, are the most commonly used techniques for detecting OP pesticides in different samples [3]–[6]. However, these commonly used methods require expensive equipment and are only applicable in laboratory settings. In comparison, immunoassays have received considerable attention as a simple, sensitive, cost-effective tool for high-throughput screening analyses in pesticide monitoring programs [7].

Immunoassays are often developed to recognize a single analyte with high specificity [8], [9], and they can also be applied for detecting various related compounds in a single test [10]–[12]. These latter immunoassays have been termed broad-selective, broad-specific, class-specific, or multi-analyte assays. Unlike a single-analyte assay, a multi-analyte assay can be used to detect the total quantity of pesticides [13]. Multi-analyte assays can also be used for pesticide monitoring before chromatographic analyses; if the total quantity of pesticides in a particular sample is less than the maximum limit, then that sample does not require further analysis [14]. To date, several investigators have developed multi-analyte enzyme-linked immunosorbent assays (ELISAs) for the detection of OP pesticides [14]–[16]. However, the ELISA detection method depends on a laboratory platform and requires a relative long assay time.

Colloidal gold has been introduced into immunochemistry and their particles could replace the enzyme to label antibody, and deposited into the conjugate pad. The capture line (test line) is hapten conjugated to a carrier protein immobilized on the membrane. Analytes in samples will compete with antigen immobilized on the membrane to bind to the colloidal gold labeled antibody. The more analytes present in the sample, the more effectively it will be able to block the capture of colloidal gold labeled antibodies. An increase in the amount of analytes in samples will result in a decrease in signal in the test line zone. Therefore, colloidal gold-conjugated immunochromatographic assays (ICAs) have been developed as a rapid and simple test for detecting chemicals in non-laboratory sites, and they have been increasingly applied in various research fields [17]–[22], including for the detection of pesticides [23]–[25]. However, few multi-analyte ICAs have been developed for the detection of OP pesticides in agricultural products.

In this study, a sensitive monoclonal antibody (C8/D3) that can recognize eight OPs was produced and used to develop an ELISA and a colloidal gold ICA for the determination of OP pesticides in agricultural products. The ELSIA showed higher sensitivity than ICA, but the ICA has advantages over ELISA methods with regard to factors such as the testing procedure, testing time, and matrix interferences, among others.

## Materials and Methods

### Reagents and Equipment

Parathion-methyl, chlorpyrifos-methyl, azinphos-methyl, dimethoate, fenitrooxon, EPN, paraoxon-ethyl, paraoxon-methyl, dicapthon, cyanophos, and famphur were purchased from Dr. Ehrensorfer (Germany). Other pesticide standards were provided by the Jiangsu Pesticide Research Institute (China). Trisodium citrate and methanol were of analytical grade. Tetramethylbenzidine (TMB), Triton x-100 and PEG 20000 were purchased from Sigma-Aldrich (USA). Horseradish peroxidase (HRP)-labeled goat anti-mouse IgG was obtained from Sino-American Biotechnology Co. (Dalian, China). Rabbit anti-mouse IgG was obtained from the Henan Provincial Key Laboratory for Animal Immunology (Henan Academy of Agriculture Sciences, China). Nitrocellulose (NC) membranes, glass-fiber conjugate pads, sample pads, and absorbent pads were produced by Millipore (USA). The BALB/c mice were obtained from the Center of Comparative Medicine of Yangzhou University (Yangzhou, China). All animals used in this study, and animal experiments, were approved by Department of Science and Technology of Jiangsu Province. The license number was SYXK (SU) 2010-0005.

The XYZ-3000 dispensing platform, guillotine cutter (Model: CM 4000) and TSR3000 membrane strip reader were supplied by BioDot (Irvine, CA, USA). Test lines were scanned using a BioDot TSR3000 Membrane Strip Reader (BioDot, CA, USA). The centrifuge (3K30) was purchased from the Sigma-Aldrich Corporation (St. Louis, USA). The protein A/G Spin Kit was purchased from the Thermo Electron Corporation (USA). The CELLine CL 350 was supplied by the Integar Biosciences Corporation (Switzerland). The transmission electron microscope (H-7650) was purchased from the Hitachi Corporation (Japan). The ultraviolet spectrophotometer (ND-1000) was purchased from the NanoDrop Corporation (USA).

### Preparation of Antigens

**Figure 1:**
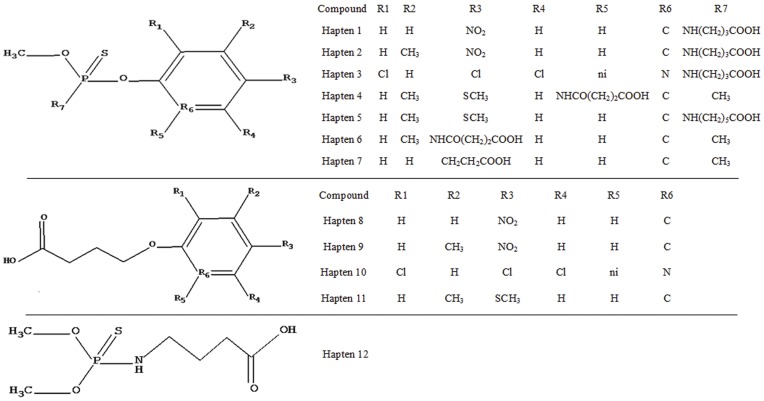
Molecular structures of the haptens. Hapten 1 was used as immunizing hapten and homologous coating hapten, hapten 2 to 12 were used as heterogenous coating hapten candidate. ni: no atom.

Figure 1 presents the structures of the 12 haptens used in the current study. The preparation of the haptens (haptens 1 to 12) and the antigens (haptens 1- to 12-OVA) has been described previously [26]–[28]. According to the active ester methodology described previously [29], hapten 1 was conjugated with BSA to generate the immunogen (hapten 1-BSA).

### Preparation of the Monoclonal Antibody (MAb)

Five 6-week-old BALB/c female mice were immunized with the H1-BSA conjugate; the immunizing strategy and the cell fusion procedures were performed as described previously [30]. When hybridomas were grown to approximately 30% confluence in culture plate wells, the culture supernatants were screened using the coating antigen hapten 1-OVA for the presence of antibodies that could recognize parathion-methyl, parathion, and fenitrothion. These three OP pesticides were chosen because they have structures more similar to the immunizing hapten than other OP pesticides and because previous studies [2], [10], [31]–[33] have demonstrated that cross-reactivity (CR) often occurs among these three pesticides. The selected hybridomas were subcloned using limiting dilution. Stable antibody-producing clones were efficiently cultivated by CELLine CL 350 according to the manufacturer’s protocol. The MAb was purified using the protein A/G Spin Kit according to the manufacturer’s protocol.

### Indirect Competitive ELISA Protocol

The concentrations of antibody and coating antigen were optimized by checkerboard titration. The ic-ELISA was performed as follow. Microtiter plates were coated with optimized concentrations of hapten-OVA in carbonate–bicarbonate buffer (50 mM, pH 9.6) by incubating for 2 h. Plates were then blocked by incubating with 1% gelatin in PBS (120 µL well^–1^) for 1.5 h. Aliquots of 25 µL well^–1^ of analyte dissolved in working solution and 25 µL well^–1^ of the supernatant (cultivated by CELLine CL 350) diluted with working solution were added to the blocked plate. After incubating for 1 h, 50 µL well^–1^ of diluted (1/20,000) goat anti-mouse IgG-HRP was added to the plate. The mixture was incubated for 1 h, followed by the addition of 50 µL well^–1^ of a TMB solution (100 µL of 1% hydrogen peroxide and 400 µL of 0.6% TMB in dimethyl sulfoxide added to 25 mL of citrate acetate buffer, pH 5.5). After incubating for 10 min, the reaction was stopped by adding 25 µL of 2 M H_2_SO_4_ and the solution absorbance was measured at 450 nm. All incubations were performed at 37°C and the plates were washed five times with PBST (10 mM PBS containing 0.05% Tween 20, pH 7.4) after each incubation, unless otherwise specified.

### Optimization of the ELSIA

The selection of coating antigens was performed by indirect ELISA and Indirect competitive ELISA. First, Twelve antigens (hapten 1 to 12-OVA) that were able to conjugate with the MAb were screened out by indirect ELISA; the indirect ELISA was run at the same time as the indirect competitive ELISA without addition of analyte. Second, the coating antigens that were screened out in the first step were selected by indirect competitive ELISA, and then the best coating antigen was confirmed based on the sensitivity of the ELISA.

The working solutions, which contained a series of pH values (5.0 ∼ 9.6) and ionic strengths (0 ∼ 3.2 M) were used to dilute the parathion-methyl, parathion, and fenitrothion standards and were tested using the established ELISA. The tested working solution that resulted in the best sensitivity for the ELISA was selected as the optimized working solution.

### Preparation of Colloidal Gold

Several investigators have reported that the average particle diameter of 40 nm colloidal gold was the optimal particle size for most diagnostic applications because of its trade-off between the required visibility and steric hindrance [34]–[36]. Therefore, in this study, nanometer colloidal gold with an average particle diameter of 40 nm was prepared according to the method described by Frens [37]. Briefly, under reflux conditions, a 100 mL solution of 0.01% gold chloride was heated to the boiling point, and 1.1 mL of 1% trisodium citrate solution was rapidly added with constant stirring. The solution was boiled for another 5 min after the color of the mixture changed to a brilliant wine red. After cooling at room temperature, the average particle diameter was evaluating using a transmission electron microscope and stored at 4°C for further study.

### Conjugation of MAb with Colloidal Gold

The MAb-gold complex was prepared according to the previously described method [38]. The pH value of the prepared colloidal gold solution was adjusted to 8.2 using 0.2 M K_2_CO_3_. 125 µL of pH adjusted colloidal gold solution was quickly added into 30 µL of serial concentrations (200, 150, 100, 50, 25, and 12.5 µg mL^−1^) of MAb. After 5 min, 125 µL of a 10% NaCl solution was added to the mixture. The OD_528_ (optical density at 528 nm) of each solution was measured using a UV-spectrophotometer in two states (stage I: before the NaCl solution was added; stage II: 60 min after the NaCl solution was added), and the experiment was performed in triplicate. In the next step, the mean decreased value between the two stages was used as the *y* axis, and the corresponding MAb concentration was used as the *x* axis to generate a curve. The point where the curve first appeared to be relatively parallel to the *x* axis was selected as the minimum quantity of MAb required to protect the gold solution from flocculation.

To conjugate MAb with colloidal gold, an amount that corresponded to 10% more than the minimum amount of MAb determined to protect the gold solution from flocculation was used. Then, the MAb solution was rapidly added to 20 mL of the colloidal gold solution (pH 8.2) with rapid stirring and incubated for 1 h. Subsequently, the mixture solution was stabilized using a 10% BSA solution (final BSA concentration was 1%) and was stirred for another 5 min. After incubation for another 1 h, the solution was centrifuged (10,000 rpm) at 4°C for 25 min, and the colorless supernatant was carefully aspirated and discarded. Subsequently, the loose sediment of the gold-labeled MAb was re-suspended using 20 mL of a 2% BSA solution (containing 0.01 M sodium borate), followed by another centrifugation (10,000 rpm at 4°C for 25 min). This procedure was repeated twice to ensure cleanup of the free (unlabeled) MAb. Finally, the loose sediment of the gold-labeled MAb was re-suspended using 4 mL of a TB solution (containing 3% BSA, 3% sucrose, 0.01 M sodium borate and 0.05% sodium azide) and stored at 4°C until needed.

### Immobilization of the Reagents

Four percent BSA was dispensed onto the glass-fiber conjugate pad using the XYZ-3000 dispensing platform, and the pad was dried at 42°C for 50 min. Subsequently, the gold-labeled MAb was dispensed onto the aforementioned glass-fiber conjugate pad using the XYZ-3000 dispensing platform, and then the pad containing gold-labeled MAb was dried again at 42°C for another 50 min. After this step, the MAb-gold conjugate pad was stored in a vacuum drying oven until needed.

The antigens and rabbit anti-mouse IgG were dispensed onto the NC membrane using the XYZ-3000 dispensing platform. The antigens were dispensed around the bottom of the NC membrane, which were used as the test line (T line), and the rabbit anti-mouse IgG was dispensed at the upper position of the NC membrane, which is used as the control line (C line). The distance between the T and the C lines was approximately 5 mm. Subsequently, the NC membrane was dried for 2 days and stored at room temperature.

### Development of the ICA Test Strip

**Figure 2:**
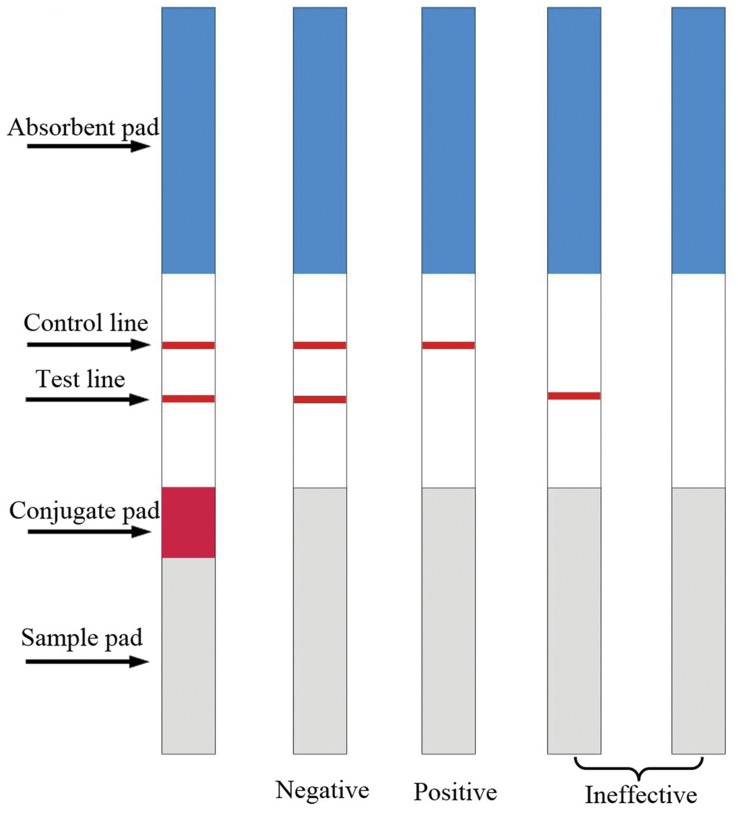
Schematic diagram of ICA test strip and result judgment. The ICA test strip was composed of the NC membrane, MAb-gold-conjugated pad, sample pad and absorbent pad, which were pasted onto an adhesive plastic backing. Result judgment was based on the color changed on the T and C line zone.

The ICA test strip was composed of the NC membrane, MAb-gold-conjugated pad, sample pad and absorbent pad, which were pasted onto an adhesive plastic backing (Figure 2). The NC membrane was pasted at the center of the backing plate. The absorbent pad was pasted by over-crossing 1 mm with the upper of the NC membrane. The mAb-gold conjugate pad was pasted by over-crossing 1 mm with the bottom of the NC membrane. The sample pad was pasted by over-crossing 2 mm with the bottom of the mAb-gold conjugate pad. Finally, the entire assembled ICA test strip was cut lengthways into 4.08 mm wide strips using a guillotine cutter.

### Test Procedure of ICA

One hundred and fifty microliters of the sample was added to the sample pad of the ICA test strip. After 5 min, the result could be evaluated by the naked eye or scanned using a membrane strip reader. When the sample solution contained the analyte, the analyte and the gold-labeled MAb formed an antigen-gold-labeled MAb complex, and the T line exhibited no color or a weaker color than the C line, which indicated that the test result was positive or weakly positive, respectively. If the sample did not contain the analyte, the T line will exhibited a similar color to the C line, which indicated that the test result was negative. The test was ineffective if no line was observed in the control line region (Figure 2).

### Optimization of the ICA Test Strip

Twelve antigens (hapten 1 to 12-OVA) were dispensed around the bottom of the NC membrane (nominal pore size: 8 µm), which was used as the T line. The antigens that conjugated with the gold-labeled MAb were first screened out, and then the best antigen was confirmed based on the sensitivity of the ICA. Four types of NC membranes (Nominal pore size: 6, 8, 10, and 12 µm) were used in this study, and the best type of NC membrane was selected based on the sensitivity and test time.

### Optimization of the Assay Conditions

The working solutions, which contained a series of pH values (5.0 ∼ 9.6), ionic strengths (0 ∼ 3.2 M), Triton x-100 (0 ∼ 1%) and polyethylene glycol (PEG) 20000 (0 ∼ 2%), were used to dilute the parathion-methyl standard and were tested using the established ICA test strip. The tested working solution that resulted in the best sensitivity for the ICA test strip was selected as the optimized working solution. The optimized working solution was allowed to migrate on the test strip, and the T line was scanned using the strip reader at different times (3, 5, 10, 20, 30, 40 and 50 min after the working solution was added). The best reading time was determined based on the stability of the G/Peak – ROD value.

### Sensitivity of the ELISA and ICA Test Strip

A series of concentrations (ELISA: 0.1∼10, 000 ng mL^−1^; ICA: 0.5 ∼ 16, 384 ng mL^−1^) of 23 analyte standards were prepared using the optimized working solution and tested using the ELISA and ICA test strip. In ICA study, the results were scanned by using a membrane strip reader, repeated three times. The average of the scanned values (ELISA: OD_450_; ICA: G/Peak - ROD) and the concentration of the analytes were used to establish the inhibition curves. Subsequently, the inhibition curves were converted into calibration curves. In the calibration curves, the binding (B/B_0_) was described as the vertical coordinate axis (*y*), and the logarithms of the analyte concentrations were designated as the abscissa axis (*x*). From these curves, the regression equations were obtained, and the IC_50_ (concentration at which binding of the antibody to the coating antigen on test line is inhibited by 50%) and limit of detection (LOD, IC_10_) were calculated.

### Cross-reactivity (CR) Studies

To evaluate the selectivity of the ELISA and ICA, 23 OP pesticides were evaluated using the established ELISA and ICA. The cross-reactivity, CR, was calculated as follows: CR = [IC_50_ (parathion-methyl)/IC_50_ (compound)] × 100%. Here, the CR of parathion-methyl was defined as 100%.

### Accuracy (Analysis of Spiked Agricultural Samples)

Three different agricultural samples (Chinese cabbage, Apple, and Greengrocery) were chosen to evaluate the performance of the multi-analyte ELISA and ICA. Chinese cabbage, Apple, and Greengrocery were bought from local markets, before the spiking and recovery studies, each test sample was verified to not contain parathion-methyl, parathion, and fenitrothion by gas chromatography. All of the samples were spiked with known concentrations of parathion-methyl, parathion, and fenitrothion in methanol.

All samples were cut into pieces and homogenized. The spiked samples were thoroughly mixed and allowed to stand at room temperature for 1 h. The sample pretreatment procedure was as follows. Acetonitrile extraction solvent (10 mL) was added to 10 g of homogenized sample, and the mixture was oscillated for 1 min with a vortex mixer. Then, 1.5 g of sodium chloride and 4 g of sodium sulfate were added, followed by oscillation for 3 min. After centrifugation (5 min, 4000 rpm), 1 mL of the supernatant was transferred and evaporated at 40°C under a nitrogen stream. The dry residue was redissolved and diluted using the optimized working solution before analysis (ELISA and ICA).

## Results and Discussion

### Production of MAb

A hybridoma cell line (C8/D3) that stably produced a monoclonal antibody (MAb) against parathion-methyl, parathion, and fenitrothion was selected from the indirect competitive ELISA and stored in China Center for Type Culture Collection, the certificate number is C201279.

### The Optimized ELISA

Four coating antigens (haptens 1, 2, 8, 9), each containing a nitro-aromatic group, were selected by indirect ELISA for their ability to conjugate with the MAb (C8/D3). These results demonstrated that the structure of the nitro-aromatic group, and its position relative to the hapten, has a very important role in the hapten’s reaction with the MAb. The sensitivity of the four antigens decreased in the following order: hapten 9> hapten 8> hapten 2> hapten 1 (Figure S1). Hapten 9-OVA was superior to the other antigens in terms of sensitivity when used as a coating antigen. Based on the IC_50_ values of parathion-methyl, parathion, and fenitrothion, the optimal ironic strength and pH of working solution was 0.4 M and 9.0, respectively (Figure S2 and S3). And 5% (V/V) methanol was used as cosolvent to improve analyte solubility. In conclusion, the optimized working solution was 0.4 M, pH 9.0 PBS buffer (containing 5% MeOH).

### Optimal Amount of mAb for Colloidal Gold

**Figure 3:**
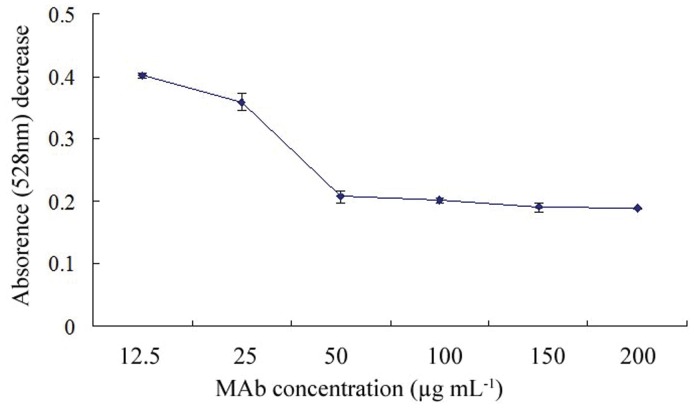
Flocculation curves. Each point on the y axis represents the mean D-value of absorbance (528 nm) in two stages (for details, see materials and methods), and the x axis values are the concentration of the mAb (n = 3).

The minimal amount of MAb was determined using a salt-induced precipitation method, and the result is presented in Figure 3. It is clear that as the MAb concentration increased, the corresponding average OD-value of the absorbance in two stages exhibited an evident decrease. Furthermore, when the MAb concentration exceeded 100 µg mL^−1^, the curve became approximately flat. Therefore, the optimal MAb concentration conjugated to colloidal gold (40 nm) solution was determined to be 110 µg mL^−1^.

### The Optimized ICA Test Strip

Four antigens (hapten 1, 2, 8, 9-OVA) were selected based on their ability to conjugate with the gold-labeled MAb (C8/D3). The sensitivity of the four antigens decreased in the following order: hapten 1-OVA>hapten 2-OVA>hapten 8-OVA>hapten 9-OVA (Table S1). Hapten 9-OVA was superior to the other antigens in terms of sensitivity when dispensed onto the NC membrane as a T line. These results suggest that using an appropriate antigen can enhance the sensitivity of the multi-analyte ICA, and the results are consistent with the ELISA measurements.

The NC membrane type is one of the most significant factors that influence the protein binding levels, sensitivity and testing time of an ICA test strip. In this study, four types of NC membranes (nominal pore sizes: 6, 8, 10, and 12 µm) were used to produce the ICA test strips. As the pore size increased, the testing time decreased and the sensitivity increased, which are good factors for a rapid test. However, the binding level decreased with increasing pore size, and there was an uneven line in the T line region for the 12 µm pore size NC membrane (Table S2). Therefore, the 10 µm pore size NC membrane was selected as the best membrane and used in the future study.

As a rapid detection tool, the result of the negative test should show visible and similar two lines (T and C line) on NC membrane. In this study, the H9-OVA was prepared in normal saline by serial dilutions ranging from 0.075 mg mL^−1^ to 1.2 mg mL^−1^. When the concentration of H9-OVA was equal or greater than 0.3 mg mL^−1^, the T line would show visible signal, but the high concentration (greater than 0.3 mg mL^−1^) of H9-OVA was used, the more parathion-methyl was needed in the sample to complete with H9-OVA. So, in this study, 0.3 mg mL^−1^ of H9-OVA solution was used to dispense on the NC membrane, and used as the test line. And the Rabbit anti-mouse IgG was prepared in normal saline solution by serial dilutions ranging from 0.2 mg mL^−1^ to 1.6 mg mL^−1^. When the concentration of Rabbit anti-mouse IgG was 0.8 mg mL^−1^, the C line would show similarly strength signal with T line. Accordingly, 0.8 mg mL^−1^ of Rabbit anti-mouse IgG solution was used to dispense on the NC membrane, and described as the control line. In order to make the two lines had the similar and easy observation width displayed on ICA strip, the dosage of H9-OVA solution and Rabbit anti-mouse IgG solution were 1.0 µL cm^−1^ in this study. Finally, 5 µL cm^−1^ gold-labeled MAb was dispensed onto the glass-fiber conjugate pad to ensure that the two lines presented a visible signal.

### The Optimized Assay Conditions

ICAs are based on antigen-antibody interactions, and the pH and ionic strength are additional factors that influence on the equilibrium constant [39]. Therefore, the pH and ionic strength of the working solution were optimized (Table S3 and S4). With the ionic strength of working solution increased, the sensitivity of ICA was improved. But when the ionic strength of the working solution is greater than 0.4 M, the gold-labeled mAb was difficultly redissolved and moved through the NC membrane with the sample solution. Therefore, the 0.4 M NaCl, pH 9.0 borate buffer was selected for the ICA because it exhibited superior sensitivity. Triton x-100 and PEG 20000 mainly affected the sensitivity, reactant flow, background color level, sharpness, and intensity of the test line. Therefore, these compounds were examined to improve the test performance; the optimized concentrations of Triton x-100 and PEG 20000 were 0.02% and 0.2%, respectively (Table S5 and S6). In conclusion, the optimized working solution was a 0.4 M NaCl, pH 9.0 borate buffer (containing 5% methanol, 0.02% Triton x-100 and 0.2% PEG 20000).

**Figure 4:**
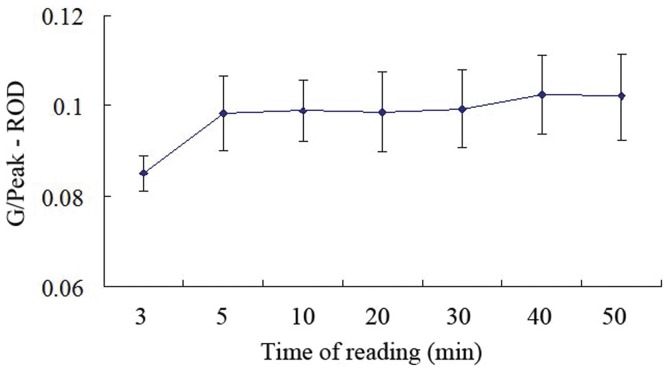
Reading time curves. Each point on the y axis represents the average value for the G/Peak - ROD, and the x axis values are the time after the addition of the working buffers (n = 3).

As shown in Figure 4, the G/Peak – ROD values increased from 0 to 5 min following the addition of the buffer. After 5 min, the G/Peak – ROD value stabilized, but after 30 min, the test strip began to desiccate and the G/Peak – ROD value began to increase. Therefore, the optimal reading time is 5 to 30 min after adding the samples.

### Sensitivity of the ELISA and ICA

**Figure 5:**
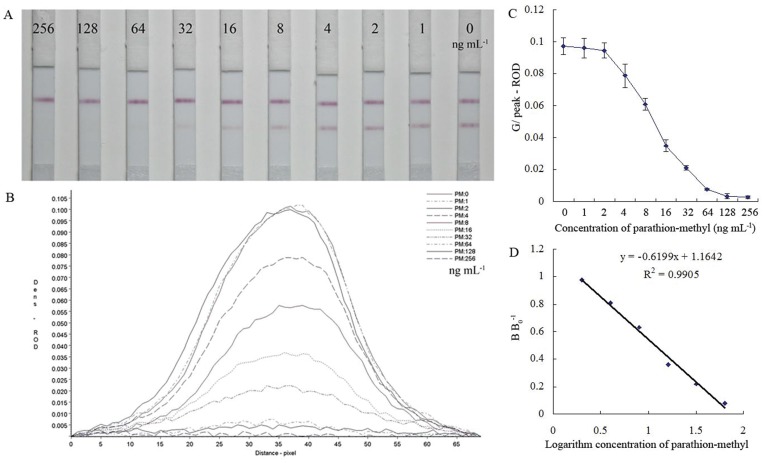
Test sensitivity. A: Concentration range of parathion-methyl standard assayed by the ICA test strip; B: Relative optical density (ROD) curves of parathion-methyl; C: Standard inhibition curves of parathion-methyl; D: The calibration curve from “A”; “B/B0” is binding ratio of antibody/antigen on the test line.

In total, 23 OP pesticides were evaluated using the ICA and ELISA methods, and the results are presented in Table 1. The IC_50_ values ranged from 11.8 to 470.4 ng mL^−1^ for the eight OP pesticides (parathion-methyl, parathion, fenitrothion, EPN, cyanophos, paraoxon-methyl, paraoxon-ethyl, and fenitrooxon), and the LOD (IC_10_) ranged from 2.7 to 100.2 ng mL^−1^ for the eight OP pesticides in the ICA test. The IC_50_ values of the ELISA ranged from 3.7 to 162.2 ng mL^–1^ for the eight OP pesticides, and the LOD (IC_10_) ranged from 0.6 to 14.2 ng mL^−1^. Furthermore, the average IC_50_ values from the ICA and ELISA tests were 183.9 and 57.1 ng mL^–1^, respectively. Figure 5 shows the immunochromatographic detection of parathion-methyl. These results indicate that the sensitivity of the newly developed ICA is lower than that of the ELISA, but this phenomenon was usually observed during the strip test [36].

**Table 1:** IC_50_ values and LOD (IC_10_) of a set of analogs related to parathion-methyl by multi-analyte ICA and ELISA.

		ICA		ELISA	
Compound		IC_50_ (ng mL^−1^)	IC_10_ (ng mL^−1^)	IC_50_ (ng mL^−1^)	IC_10_ (ng mL^−1^)
1	Parathion-methyl	11.8	2.7	3.7	0.6
2	Parathion	20.8	4.2	11.3	1.9
3	Fenitrothion	47.4	10.6	8.9	0.9
4	Cyanophos	71.1	15.7	20.3	2.2
5	EPN	58.6	13.3	18.3	2.8
6	Paraoxon-methyl	439.2	92.3	118.7	6.9
7	Paraoxon-ethyl	470.4	100.2	162.2	14.2
8	Fenitrooxon	352.2	71.5	113.6	10.3
9	Dicapthon	2018.1	479.4	576.6	83.2
10	Famphur	>100000	ni	58215.7	9991.8
11	Isocarbophos	>100000	ni	>100000	ni
12	Fenthion	>100000	ni	>100000	ni
13	Triazophos	>100000	ni	>100000	ni
14	Chlorpyrifos	>100000	ni	>100000	ni
15	Chlorpyrifos-methyl	>100000	ni	>100000	ni
16	Phoxim	>100000	ni	>100000	ni
17	Malathion	>100000	ni	>100000	ni
18	Phorate	>100000	ni	>100000	ni
19	Dimethoate	>100000	ni	>100000	ni
20	Acephate	>100000	ni	>100000	ni
21	Dichlorvos	>100000	ni	>100000	ni
22	Tolclofos-methyl	>100000	ni	>100000	ni
23	Azinphose-methyl	>100000	ni	>100000	ni
Mean for 1–8		183.9	38.8	57.1	5.0
CV (%) for 1–8		108.5	107.5	110.0	100.4

ni means no inhibition by a pesticide.

### Cross-reactivity

The selectivity of the ICA and ELISA were evaluated by measuring the cross-reactivity with the 23 OP pesticides; the CRs for each compound are summarized in Table 2. These results indicated that the OP pesticides with CR are mainly the eight OP pesticides, and the CRs for the ICA were similar to those from the ELISA. Therefore, the developed ELISA and ICA had specific selectivity for the eight OP pesticides.

**Table 2:** Cross-reactivity (CR) of a set of analogs related to parathion-methyl by multi-analyte ICA and ELISA.

	CR (%)	
Compound	ICA	ELISA
Parathion-methyl	100	100
Parathion	56.7	32.9
Fenitrothion	24.9	41.4
Cyanophos	16.6	18.2
EPN	20.1	20.2
Paraoxon-methyl	2.7	3.1
Paraoxon-ethyl	2.5	2.3
Fenitrooxon	3.4	3.3
Dicapthon	0.6	0.6
Famphur	<0.01	0.006
Isocarbophos	<0.01	<0.003
Fenthion	<0.01	<0.003
Triazophos	<0.01	<0.003
Chlorpyrifos	<0.01	<0.003
Chlorpyrifos-methyl	<0.01	<0.003
Phoxim	<0.01	<0.003
Malathion	<0.01	<0.003
Phorate	<0.01	<0.003
Dimethoate	<0.01	<0.003
Acephate	<0.01	<0.003
Dichlorvos	<0.01	<0.003
Tolclofos-methyl	<0.01	<0.003
Azinphose-methyl	<0.01	<0.003

### Analysis of Spiked Samples

**Figure 6:**
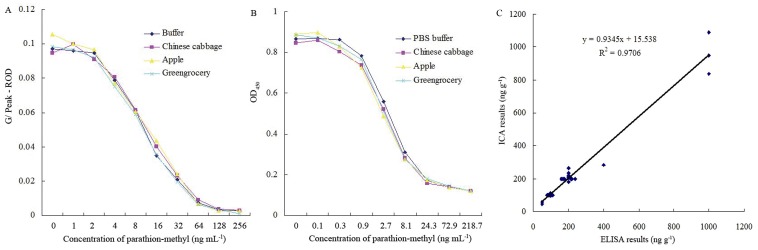
Matrix interference study and correlation of recoveries between the ICA and ELISA. A: Standard inhibition curves for parathion-methyl in the buffer and different matrices using the multi-analyte ICA, Chinese cabbage and Apple samples were diluted 5-fold, and greengrocery samples were diluted 20-fold; B: Standard inhibition curves for parathion-methyl in the buffer and different matrices using the multi-analyte ELISA, Chinese cabbage, Apple and greengrocery samples were diluted 40-fold; C: The degree of correlation between the multi-analyte ICA and ELISA for analyses of samples spiked with parathion-methyl, parathion, and fenitrothion.

**Figure 7:**
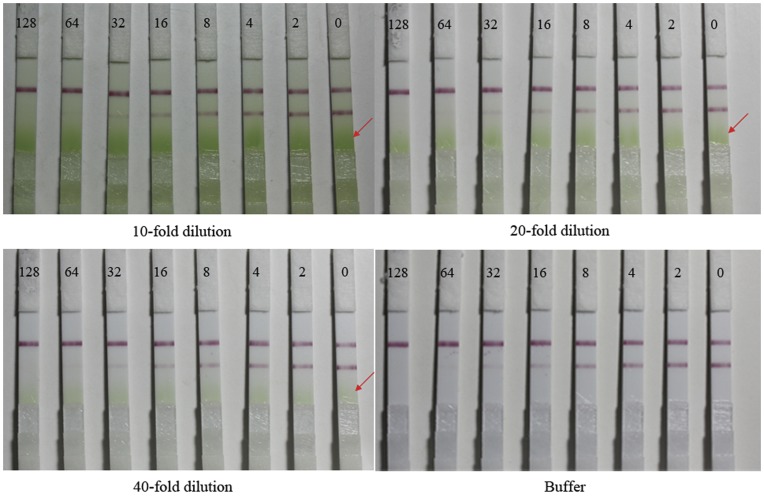
Matrix interference of greengrocery in ICA test strip. ICA test strip detection of parathion-methyl with 10-, 20-, 40-fold dilutions of greengrocery matrix interference and buffer. Standard solutions of parathion-methyl at each final concentration of 128, 64, 32, 16, 8, 4, 2 and 0 ng mL-1 (numbers across the top of strips from left to right) were tested.

One of the most common challenges in developing an immunoassay is matrix interference. Chemical compounds present in samples or sample extracts, such as humic acids, solvents, pigment, and heavy metal ions, may affect the binding between the antibody and analyte, thereby reducing the sensitivity and reliability of the immunoassay and possibly leading to false positive results [40]. Matrix interferences can be reduced in a number of ways, and dilution with buffers is a commonly used procedure [41], [42]. In this study, the effects of different dilution ratios using the optimized working buffer from 1∶1 to 1∶80 (v/v) on reducing the degree of matrix interferences were tested. These results indicated that the G/Peak–ROD values of the standard curves prepared during the matrix dilution were somewhat similar to those in the matrix free buffer when the dilution ratio was increased. During the ICA, the matrix interference was adequately removed from the Chinese cabbage and Apple samples with at least a 5-fold dilution, and the interference was removed from the greengrocery samples with at least a 20-fold dilution. However, during the ELISA, when the matrixes were diluted equal or greater than 40-fold, the standard inhibition curves prepared during the matrix dilution were somewhat similar to those in the matrix free buffer (Figure 6). Therefore, at least a 40-fold dilution was required to adequately remove the matrix interference from the Chinese cabbage, Apple, and greengrocery samples. These results indicated that the ICA has an advantage over ELISA for matrix interferences. Meanwhile, comparison of the matrix interference of Chinese cabbage and Apple with greengrocery samples in the ICA indicates that the greengrocery samples exhibited the strongest matrix interference. These observations can be explained by the fact that greengrocery is a green leafy vegetable that contains an abundance of chlorophyll; the effect of chlorophyll interference is presented in Figure 7. Figure 7 also indicated that most of the chlorophyll was centered at the bottom of the NC membrane (red signer). This result indicated that the NC membrane has the ability to separate chlorophyll from the buffer, but the NC membrane cannot completely separate the chlorophyll; therefore, the matrix inference of greengrocery samples is stronger than that of Chinese cabbage and Apple samples. The results also indicated that identifying and using materials that have the ability to separate matrix inferences are important for removing matrix inferences and improving the reliability and stability of the ICA.

The measured recoveries from the spiked samples are summarized in Table 3. The recoveries ranged from 70.6 to 131.9 for parathion-methyl, parathion, and fenitrothion. Furthermore, the CV ranged from 5.1% to 35.5%. Therefore, the recoveries of these samples were satisfactory. To confirm the accuracy and applicability of the multi-analyte ICA, the spiked samples were analyzed using ELISA. The results are shown in Table 4. The average recoveries using ELISA from all samples ranged from 79.1% to 118.1%, which were similar to those of the ICA measurements described here. Figure 6 compares the multi-analyte ICA and ELISA data. The line equation and correlation coefficient obtained from linear regression of the combined ICA and ELISA data for parathion-methyl, parathion, and fenitrothion were *y* = 0.9345*x* +15.538 and R^2^ = 0.9706, respectively. The high degree of linearity indicates that the ICA and ELISA techniques yielded comparable results.

**Table 3:** Recovery studies of samples spiked with parathion-methyl, parathion, and fenitrothion by multi-analyte ICA (*n* = 4).

Compound	Sample	Spiked (ng g^−1^)	Measured ±SD (ng g^−1^)	Recovery (%)	CV (%)
Parathion-methyl	Chinese cabbage	50	46.8±10.9	93.7	23.3
		200	206.6±10.6	103.3	5.1
	Apple	50	45.4±6.9	90.8	14.3
		200	180.1±11.0	90.1	6.1
	Greengrocery	200	218.8±25.2	109.4	11.5
		1000	949.4±51.2	94.9	5.4
Parathion	Chinese cabbage	50	44.7±16.4	89.3	36.8
		200	204.1±18.2	102.1	6.8
	Apple	50	58.5±11.0	116.9	18.8
		200	213.4±16.8	106.7	7.9
	Greengrocery	200	219.2±55.3	109.6	25.2
		1000	835.7±102.7	83.6	12.3
Fenitrothion	Chinese cabbage	100	97.4±34.6	97.4	35.5
		200	231.7±58.4	115.9	18.2
	Apple	100	110.5±7.5	110.5	6.8
		200	263.8±30.2	131.9	11.4
	Greengrocery	400	282.5±79.3	70.6	28.1
		1000	1088.8±161.7	108.9	14.9

**Table 4:** Recovery studies of samples spiked with parathion-methyl, parathion, and fenitrothion by multi-analyte ELISA (*n* = 3).

Compound	Sample	Spiked (ng g^−1^)	Measured ±SD (ng g^−1^)	Recovery (%)	CV (%)
Parathion-methyl	Chinese cabbage	100	80.1±6.5	80.1	8.1
		200	158.2±5.4	79.1	3.4
	Apple	100	89.0±8.1	89.1	9.1
		200	236.3±7.3	118.1	3.1
	Greengrocery	100	105.2±10.7	105.2	10.1
		200	215.2±21.9	107.6	10.2
Parathion	Chinese cabbage	100	86.1±13.7	86.1	15.9
		200	164.7±5.9	82.4	3.6
	Apple	100	89.0±8.9	89.0	10.1
		200	177.4±9.6	88.7	5.4
	Greengrocery	100	112.2±23.3	112.2	20.8
		200	221.2±14.7	110.0	7.4
Fenitrothion	Chinese cabbage	100	90.7±9.7	90.7	10.7
		200	172.5±10.9	86.2	6.3
	Apple	100	78.9±6.7	78.9	8.5
		200	175.4±12.1	87.7	6.9
	Greengrocery	100	89.6±13.9	89.6	15.6
		200	217.6±11.1	108.8	5.1

### Conclusions

This work presents an ELISA and a gold-labeled antibody ICA test strip for detecting OP pesticides in agricultural samples. The quantitative data indicated that the specificity and accuracy of ICA were ideal and in good agreement with the ELISA measurements. The ELISA has higher sensitivity than ICA for the detection of OP pesticides in agricultural products. But the ICA technique did not require intensive labor or expensive equipment for the sample analysis; the procedure was very simple, and the detection was completed within 5 min. In summary, the established ICA method might provide an alternative tool for the sensitive, rapid, and convenient detection of OP pesticides in agricultural products, and it has advantages over ELISA methods with regard to factors such as the testing procedure, testing time, and matrix interferences, among others.

## Supporting Information

Figure S1 **Effects of coating antigen on ELISA.** The IC_50_ values of parathion-methyl, parathion, and fenitrothion on four different coating antigen ELSIAs (n = 3).
(TIF)

Figure S2 **Effects of ionic strength on ELSIA.** Each point on the y axis represents the mean IC_50_ of parathion-methyl, parathion, and fenitrothion, and the x axis values are the concentration of NaCl (n = 3).
(TIF)

Figure S3 **Effects of pH value on ELSIA.** Each point on the y axis represents the mean IC_50_ of parathion-methyl, parathion, and fenitrothion, and the x axis values are pH values of the solution (n = 3).
(TIF)

Table S1 Effects of antigens on the gold immunochromatographic assay (n = 3).
(DOC)

Table S2 Comparison of difference NC membrane on sensitivity, test time, and T line color.
(DOC)

Table S3 Effects of ionic strength on the gold immunochromatographic assay (n = 3).
(DOC)

Table S4 Effects of pH values on the gold immunochromatographic assay (n = 3).
(DOC)

Table S5 Effects of Triton x-100 on the immunochromatographic assay (n = 3).
(DOC)

Table S6 Effects of PEG 20000 on the immunochromatographic assay (n = 3).
(DOC)
