# Supplementary figures and images for: Development of an Enzyme Linked Immunosorbent Assay and an Immunochromatographic Assay for Detection of Organophosphorus Pesticides in Different Agricultural Products
Source: PLoS One. 2012 Dec 31;7(12):e53099. doi: 10.1371/journal.pone.0053099 (PMC3534045; doi:10.1371/journal.pone.0053099)

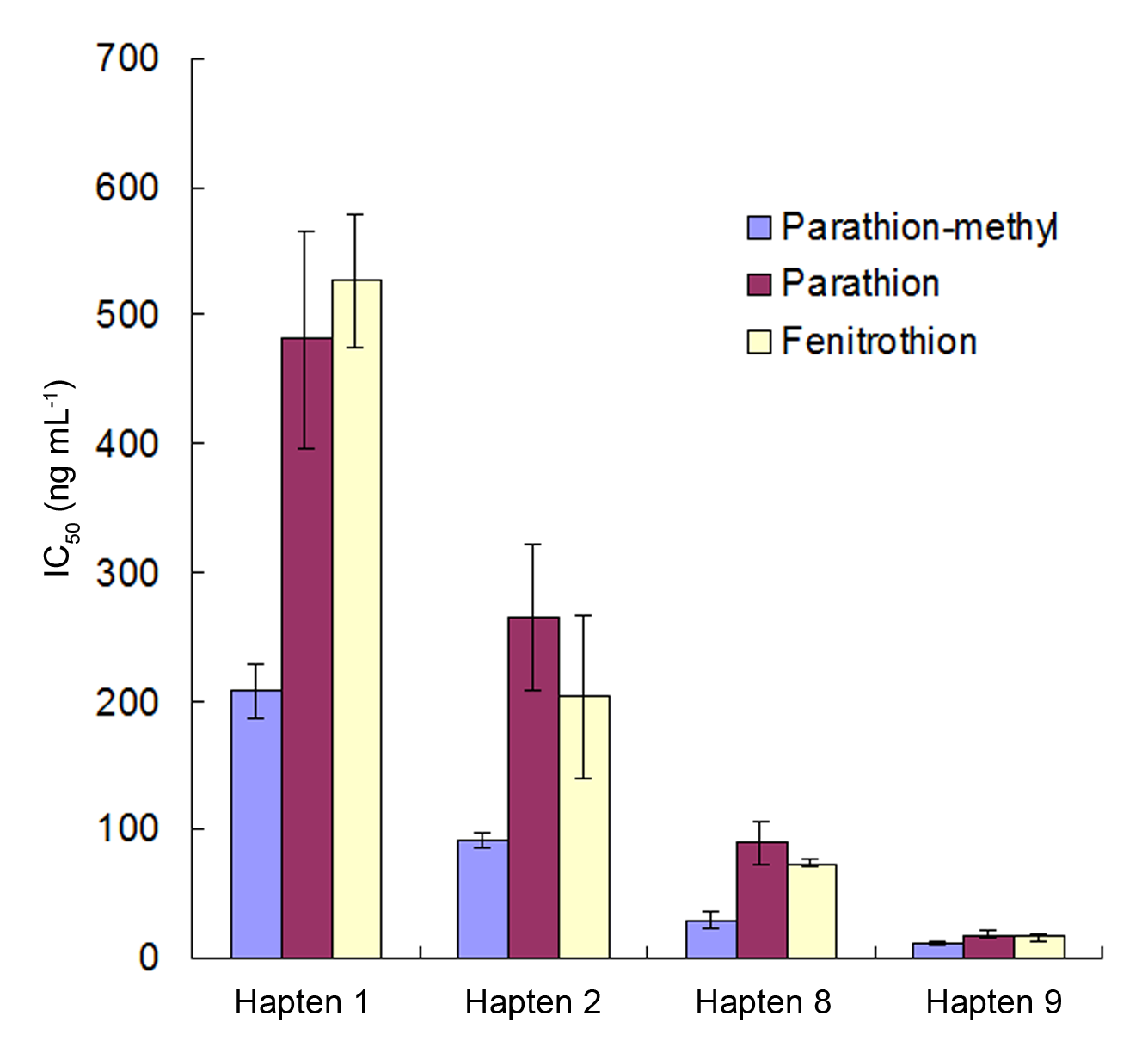

Supplement: Figure S1 — Effects of coating antigen on ELISA. The IC50 values of parathion-methyl, parathion, and fenitrothion on four different coating antigen ELSIAs (n = 3). (TIF) [file pone.0053099.s001.tif]

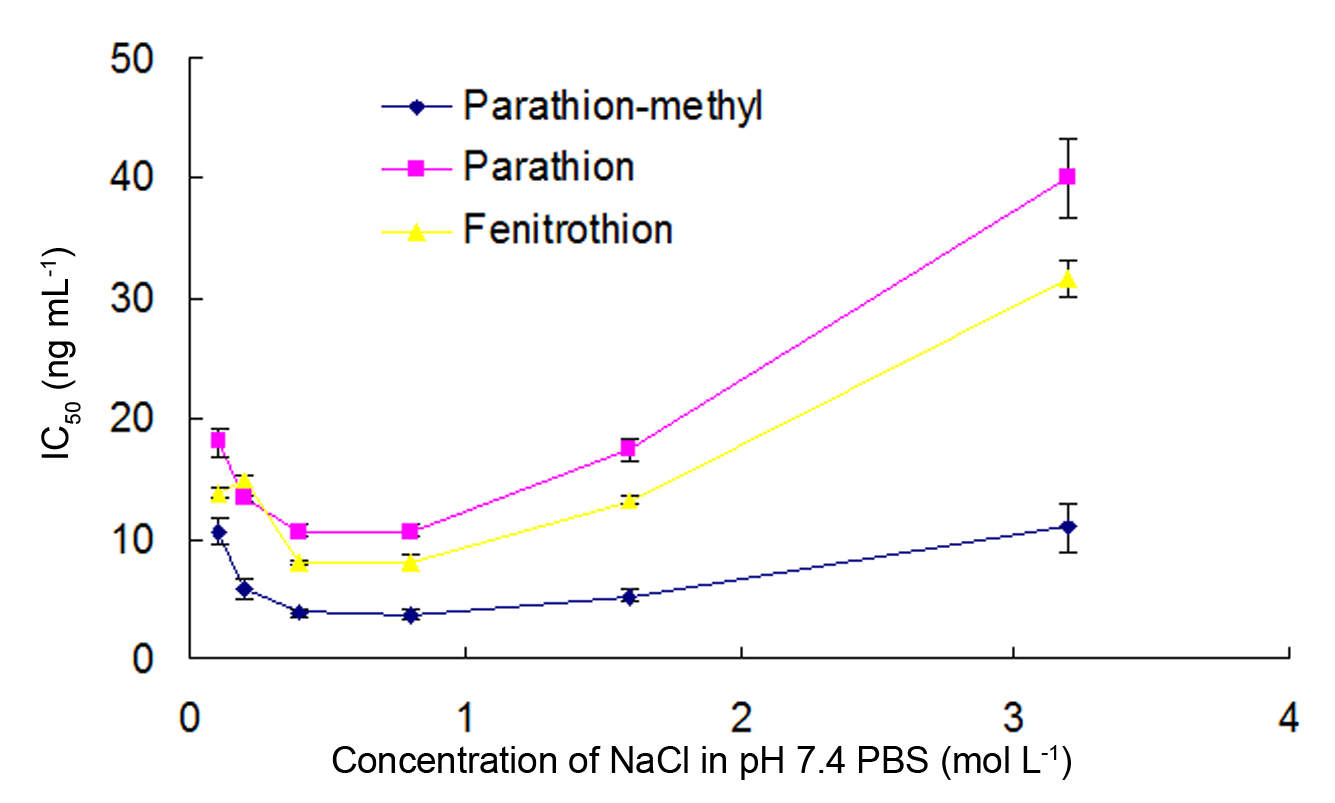

Supplement: Figure S2 — Effects of ionic strength on ELSIA. Each point on the y axis represents the mean IC50 of parathion-methyl, parathion, and fenitrothion, and the x axis values are the concentration of NaCl (n = 3). (TIF) [file pone.0053099.s002.tif]

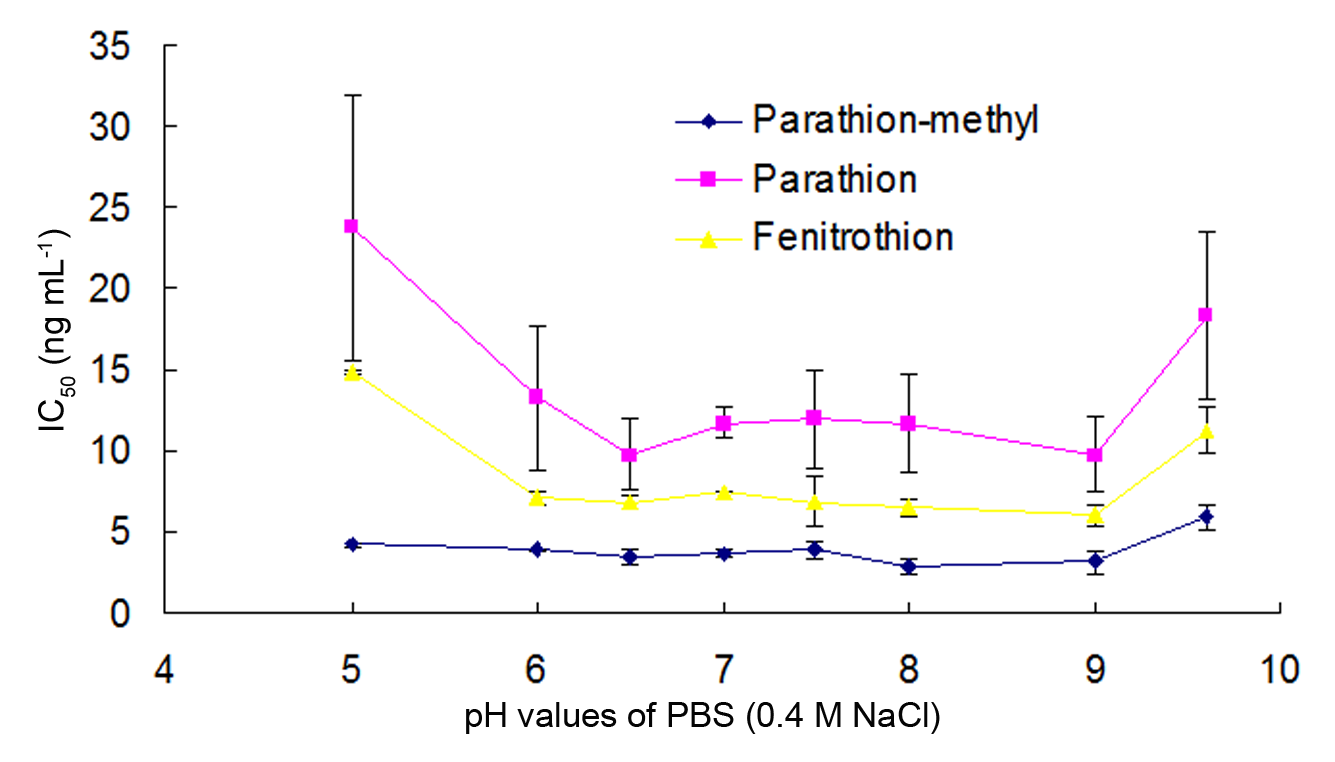

Supplement: Figure S3 — Effects of pH value on ELSIA. Each point on the y axis represents the mean IC50 of parathion-methyl, parathion, and fenitrothion, and the x axis values are pH values of the solution (n = 3). (TIF) [file pone.0053099.s003.tif]
