# Supplementary material for: Development of an Enzyme Linked Immunosorbent Assay and an Immunochromatographic Assay for Detection of Organophosphorus Pesticides in Different Agricultural Products
Source: PLoS One. 2012 Dec 31;7(12):e53099. doi: 10.1371/journal.pone.0053099 (PMC3534045; doi:10.1371/journal.pone.0053099)
Supplement: Table S1 — Effects of antigens on the gold immunochromatographic assay (n = 3). (DOC) [file pone.0053099.s004.doc]

Table S1 Effects of antigens on the gold immunochromatographic assay (n=3).

|  |  | **parathion-methyl standard concentration(µg mL^-1^)** | | | | | | |
| --- | --- | --- | --- | --- | --- | --- | --- | --- |
| **Antigens** |  | **0** | **0.3** | **0.6** | **1.25** | **2.5** | **5** | **10** |
| Hapten 1-OVA | Test line | +++ | +++ | +++ | +++ | +++ | ++ | +± |
|  | Control line | +++ | +++ | +++ | +++ | +++ | +++ | +++ |
| Hapten 2-OVA | Test line | +++ | +++ | +++ | +++ | ++ | +± | + |
|  | Control line | +++ | +++ | +++ | +++ | +++ | +++ | +++ |
| Hapten 8-OVA | Test line | +++ | +++ | ++ | +± | + | ± | ± |
|  | Control line | +++ | +++ | +++ | +++ | +++ | +++ | +++ |
| Hapten 9-OVA | Test line | +++ | ++ | + | ± | - | - | - |
|  | Control line | +++ | +++ | +++ | +++ | +++ | +++ | +++ |

+++: Red line appeared.

++±: Red line appeared but was weaker than +++.

++: Red line appeared but was weaker than ++±.

+±: Red line appeared but was weaker than++.

+: Red line appeared but was weaker than +±.

±: Red line appeared but was weaker than +.

-: Red line did not appear.
