# Supplementary material for: Development of an Enzyme Linked Immunosorbent Assay and an Immunochromatographic Assay for Detection of Organophosphorus Pesticides in Different Agricultural Products
Source: PLoS One. 2012 Dec 31;7(12):e53099. doi: 10.1371/journal.pone.0053099 (PMC3534045; doi:10.1371/journal.pone.0053099)
Supplement: Table S2 — Comparison of difference NC membrane on sensitivity, test time, and T line color. (DOC) [file pone.0053099.s005.doc]

Table S2 Comparison of difference NC membrane on sensitivity, test time, and T line color.

| **Nominal pore size** | **Sensitivity^a^** | **Test time^b^** | **T line color** |
| --- | --- | --- | --- |
| 6 μm NC membrane | 2 µg mL^-1^ | ＞8 min | even |
| 8 μm NC membrane | 2µg mL^-1^ | ＞5 min | even |
| 10 μm NC membrane | 1µg mL^-1^ | ＞5 min | even |
| 12 μm NC membrane | 1µg mL^-1^ | ＞3 min | uneven |

^a^ Concentration of parathion-methyl that can completely inhibited the conjugation of antigen and gold label-MAb.

^b^ The time of all gold label-MAb had passed NC membrane.
