# Supplementary material for: Development of an Enzyme Linked Immunosorbent Assay and an Immunochromatographic Assay for Detection of Organophosphorus Pesticides in Different Agricultural Products
Source: PLoS One. 2012 Dec 31;7(12):e53099. doi: 10.1371/journal.pone.0053099 (PMC3534045; doi:10.1371/journal.pone.0053099)
Supplement: Table S6 — Effects of PEG 20000 on the immunochromatographic assay (n = 3). (DOC) [file pone.0053099.s009.doc]

Table S6 Effects of PEG 20000 on the immunochromatographic assay (n=3).

|  |  | **parathion-methyl standard concentration(µg mL^-1^)** | | | | | | | |
| --- | --- | --- | --- | --- | --- | --- | --- | --- | --- |
| **Concentration of PEG 20000** |  | **0** | **0.03** | **0.06** | **0.125** | **0.25** | **0.5** | **1** | **2** |
| 0 | Test line | +++ | +± | + | ± | ± | - | - | - |
|  | Control line | +++ | +++ | +++ | +++ | +++ | +++ | +++ | +++ |
| 0.02% | Test line | +++ | +± | + | ± | - | - | - | - |
|  | Control line | +++ | +++ | +++ | +++ | +++ | +++ | +++ | +++ |
| 0.05% | Test line | +++ | +± | + | ± | - | - | - | - |
|  | Control line | +++ | +++ | +++ | +++ | +++ | +++ | +++ | +++ |
| 0.1% | Test line | +++ | + | ± | - | - | - | - | - |
|  | Control line | +++ | +++ | +++ | +++ | +++ | +++ | +++ | +++ |
| 0.2% | Test line | +++ | ± | ± | - | - | - | - | - |
|  | Control line | +++ | +++ | +++ | +++ | +++ | +++ | +++ | +++ |
| 0.5% | Test line | +++ | +± | + | ± | - | - | - | - |
|  | Control line | +++ | +++ | +++ | +++ | +++ | +++ | +++ | +++ |
| 1% | Test line | +++ | +± | + | ± | - | - | - | - |
|  | Control line | +++ | +++ | +++ | +++ | +++ | +++ | +++ | +++ |

+++: Red line appeared.

++±: Red line appeared but was weaker than +++.

++: Red line appeared but was weaker than ++±.

+±: Red line appeared but was weaker than++.

+: Red line appeared but was weaker than +±.

±: Red line appeared but was weaker than +.

-: Red line did not appear.
